# Congenital lamellar ichthyosis in Tunisia is caused by a founder nonsense mutation in the *TGM1* gene

**DOI:** 10.1007/s11033-012-2333-1

**Published:** 2012-11-29

**Authors:** Nacim Louhichi, Ikhlass Hadjsalem, Slaheddine Marrakchi, Fatma Trabelsi, Abderrahmen Masmoudi, Hamida Turki, Faiza Fakhfakh

**Affiliations:** 1 Human Molecular Genetic Laboratory, Faculty of Medicine of Sfax, Avenue Magida Boulila, 3029 Sfax, Tunisia; 2 Department of Dermatology, Hedi Chaker Hospital, Sfax, Tunisia

**Keywords:** *TGM1* gene, Nonsense mutation, Founder effect, c.788G>A, p.W263X, Congenital lamellar ichthyosis

## Abstract

Lamellar ichthyosis (LI, MIM# 242300) is a severe autosomal recessive genodermatosis present at birth in the form of collodion membrane covering the neonate. Mutations in the *TGM1* gene encoding transglutaminase-1 are a major cause of LI. In this study molecular analysis of two LI Tunisian patients revealed a common nonsense c.788G>A mutation in *TGM1* gene. The identification of a cluster of LI pedigrees carrying the c.788G>A mutation in a specific area raises the question of the origin of this mutation from a common ancestor. We carried out a haplotype-based analysis by way of genotyping 4 microsatellite markers and 8 SNPs flanking and within the *TGM1* gene spanning a region of 6 Mb. Haplotype reconstruction from genotypes of all members of the affected pedigrees indicated that all carriers for the mutation c.788G>A harbored the same haplotype, indicating common ancestor. The finding of a founder effect in a rare disease is essential for the genetic diagnosis and the genetic counselling of affected LI pedigrees in Tunisia.

## Introduction

Autosomal recessive congenital ichthyosis (ARCI) is a rare, clinically and genetically heterogeneous group of cornification diseases. It defines three clinical subtypes which include the spectrum of lamellar ichthyosis (LI; OMIM 242300), congenital ichthyosiform erythroderma (CIE; OMIM 242100) and recently harlequin ichthyosis (HI; OMIM 242500) [1]. The ARCI is characterized by epidermal scaling, often collodion membrane at birth, ectropion, eclabium, alopecia, palmar-plantar hyperkeratosis, hypohidrosis and/or variable erythema [2].

Over the last decade at least six different genes have been implicated in the etiology of ARCI, all of which encode proteins essential for the formation of the skin barrier: *TGM1* [3, 4], *ABCA12* [5, 6], two lipoxygenase genes (*ALOXE3* and *ALOX12B*) [7, 8], ichthyin (*NIPAL4*) [9] and CYP4F2 homolog (*FLJ39501*) [10].

Lamellar ichthyosis (LI) constitutes a rare disease but its prevalence may increase in population which has a high consanguinity owing to fonder effect [11–13]. The most common cause of LI/CIE is inactivating mutations in the *TGM1* gene, which encodes transglutaminase-1, an enzyme that crosslinks proteins in the cornified cell envelope [4, 14].

The *TGM1* gene has 14,095 bp, including 2,454 bp of coding sequence, and spans 15 exons (GenBankNM_000359.2). It encodes for the transglutaminase-1 (TGase-1) enzyme, which has 817 amino acid residues and a molecular weight of ~89 kDa (GenBank NM_000359.2) [15, 16]. It is a catalytic membrane-bound enzyme involved in the formation of the epidermal cornified cell envelope, which acts as a mechanical barrier to protect against water loss and infectious agents [14]. Up to now, more than 130 *TGM1* different pathogenic mutations have been reported in the literature associated with LI/CIE phenotypic spectrum in patients from diverse ethnic backgrounds [1]. *TGM1* mutations include missense, nonsense, and splice site mutations. The majority are single-base changes; rarely, insertions or deletions are found.

This study presents detailed molecular analysis of lamellar ichthyosis in two Tunisian patients belonging to two consanguineous families. They present a common nonsense mutation c.788G>A in *TGM1* gene. Since it is the only mutation described to be responsible for the LI in Tunisia, we confirmed a founder effect using polymorphic microsatellite markers.

## Patients and methods

Two patients belonging to two Tunisian families were born to healthy consanguineous parents. Genealogical investigation was performed and excluded any close relation between both families. Five additional healthy siblings and the parents were also recruited. Informed consent was obtained from patients and control individuals in accordance with the ethics committee of Hedi Chaker Hospital (Sfax, Tunisia). The diagnosis of LI was made on the basis of clinical manifestation; a detailed medical and dermatologic history was obtained from the affected persons. They were examined more than one time over the period of 10 years so that potential changes in phenotype could be evaluated. Blood samples were collected from family members and healthy individuals. Genomic DNA was extracted from the whole blood following a standard phenol–chloroform method [17].

## Mutation analysis for *TGM1* gene

The 15 exons and flanking intron region of the *TGM1* gene were tested for mutation in the ARCI patients by sequence analysis. Following DNA extraction, the coding regions and intron–exon boundaries of the *TGM1* gene were amplified by polymerase chain reaction (PCR) using generated primers covering the entire coding region (Table 1). PCR was carried out on 50 μl volume samples, in a GeneAmp^®^ PCR system 9700 (Applied Biosystems, Foster City, CA, USA). Each sample contains 100 ng of genomic DNA, 0.4 μM of each primer, 0.2 mM of dNTPs, 1 IU of Amplitaq and 10× PCR Buffer II in a final concentration of 1 × 1.5 mM of MgCl_2_. The mixture was denatured during 10 min at 95 °C and then followed by 35 cycles: denaturing at 95 °C for 45 s, annealing at 65/60 °C for 45 s and an extension at 72 °C for 45 s; with a final extension at 72 °C for 7 min. Thereafter, the purified amplicons were directly sequenced using a dye terminator cycle sequencing kit V1.1 with an ABI sequence analyser 3100 avant (Applied Biosystems, Foster City, CA, USA) according to the manufacturer’s recommendations.

**Table 1:** Primer pairs of *TGM 1* gene used for detection of mutation by DNA sequencing

Exon	Forward	Reverse	PCR size (bp)
TGM1-Ex1	5′CTAAGCCCCCAGACCTCAC3′	5′CAGGGAAAGCAGAGTCTTGG3′	386
TGM1-Ex2	5′GTGGGATTGTTTCGGTCATC3′	5′GCTGAGTCTCTGGTCCCATTAA3′	498
TGM1-Ex3	5′GAGACTCAGCCTGGCATTTC3′	5′CAGGGAGGAGCCTAAAGACC3′	379
TGM1-Ex4	5′GGGACATACACAGTGGCTCATA3′	5′GAAGCCCTTCCCTGTCTTTC3′	420
TGM1-Ex5	5′GCAGAACTGGCCAGAAGTAGGT3′	5′ACCCACCCCAGCTCCTCT3′	246
TGM1-Ex6	5′GAGCAGGGTGCTGGCCTA3′	5′CCAGAAAGGGCAGGAGGAG3′	250
TGM1-Ex7	5′TGTCTGGATCCTGAGGCATTTAG3′	5′CCCTGCACTGTAGCCACATCT3′	405
TGM1-Ex8	5′CCAGCTTGGCAATCCTAGTT3′	5′AAGTTCCTGGATGGACATGG3′	395
TGM1-Ex9	5′ATGGTGACCTGAGCTTTGGA3′	5′CCAGCACTGACACTCTGGACT3′	296
TGM1-Ex10	5′CCCTGTGTGGACCTTACCC3′	5′GGTCAGTCAGCGGTGAAGTT3′	247
TGM1-Ex11	5′TCTGGCTCGTCTTGGAAAGT3′	5′AAGCACTTGGCAGGAACACT3′	380
TGM1-Ex12	5′ACAACAAGTGTTCCTGCCAAGT3′	5′ACTGAGGCCTGCTTCCCTAC3′	481
TGM1-Ex13	5′GCCTGTAAGTGCTCCTTACCC3′	5′GCCCACCTCTGATGTCCTTA3′	386
TGM1-Ex14	5′CTCTCTGGTGCAGTGTACGG3′	5′ACAGAGAGGGAGCAAAGCTG3′	300
TGM1-Ex15	5′CTCCTGCTTCTTCCTCCTGA3′	5′GTGTGGCATGGACTGAAAGA3′	558

## Microsatellites and SNPs screening

For the haplotypic analyses, we screened four microsatellite markers D14S283, D14S275, D14S72 and D14S80. They occur in the 5′ and 3′ flanking region of the *TGM1* gene and reported as highly polymorphic STR [12]. Sequences of the primers for genetic markers were obtained from UNISTS (http://www.ncbi.nlm.nih.gov/unists).

Fluorescent dye-labeled microsatellite markers were genotyped for all the family members. We used the True Allele PCR Premix (Applied Biosystems, Foster City, CA) for PCR reactions according to the manufacturer’s instructions. Fluorescently-labelled alleles were analysed on an ABI PRISM 3100-Avant automated Genetic Analyser (Applied Biosystems, Foster City, CA). Genotypes were determined using the GenScan™ software (Applied Biosystems, Foster City, CA). The haplotype co-segregating with the disease was derived from the segregation of markers within the two pedigrees.

## Results

### Clinical analysis of ARCI patients

We identified two unrelated Tunisian families with two LI patients (MAS and NC). The healthy parents were consanguineous and originated from south Tunisia. Clinical data indicated that the initial presentation was a congenital onset of the disease with a history of collodion presentation and heat intolerance. MAS is a 14-year old son, second child of healthy parents who are first cousins, was born at term. Since his birth he developed a severe lamellar ichthyosis. He had one brother died for unknown reasons. NC is a 21-year old girl was referred to the dermatology department at Hedi Chaker Hospital in Sfax for a severe lamellar ichthyosis since birth, she is the first child of healthy consanguineous parents.

**Fig. 1:**
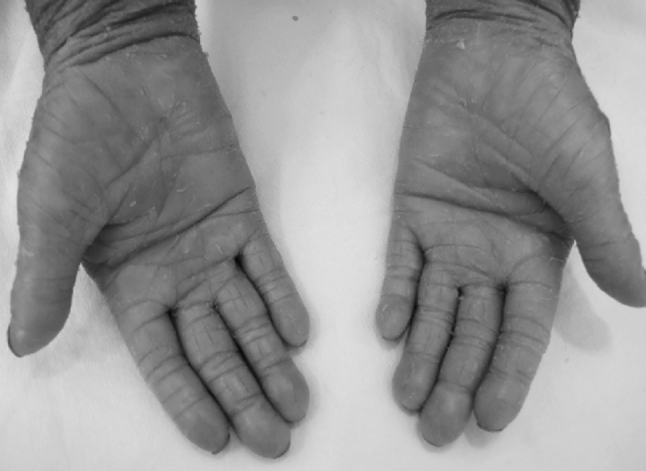
NC patient’s hands showing palmar keratoderma and brachydactyly

The two patients (MAS and NC) showed a normal intellect. No evidence of congenital hypothyroidism or sensitivity to gluten usually associated to LI Tunisian phenotype was noted. The main clinical features of the two patients are summarized in Table 2 (Table 2; Fig. 1).

**Table 2:** Clinical data of LI patients

Patient 1 (MAS)	Patient 2 (NC)
Collodion baby	Collodion baby
Erythematous skin with large thick scaly	Episodes of bullous detachment in childhood
Brownish scales of the limbs	Erythematous skin
Everted lips	Brownish scales of the limbs
Adhered ears	No everted lips
Ectropion eyelid, broad nasal base	Adhered ears
Palmoplantar keratoderma	Ectropion eyelid
Brachydactyly and tapering fingernails	Palmoplantar keratoderma
Frontal hairline rarefaction	Brachydactyly (Fig. 1) and tapering fingernails
	Frontal alopecia

### One point mutation within *TGM1* gene revealed by nucleotide sequencing

**Fig. 2:**
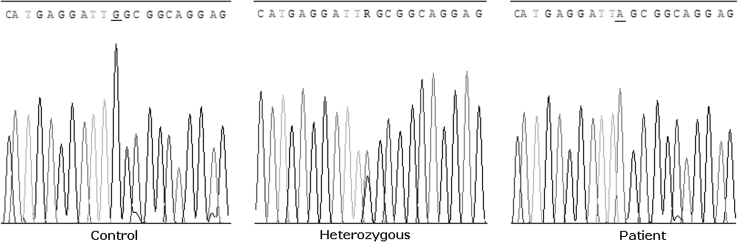
Sequence chromatograms of the TGM1 gene in the region of the c.788G>A mutation, showing a control, carrier and mutant subject. Nucleotide variations are *underlined*

Mutation screening of the *TGM1* coding region and intron–exon boundaries revealed the c.788G>A nonsense mutation (g.3139 G>A) in the two patients at homozygous state associated with a severe LI phenotype (Fig. 2). It occurs in exon 5 and results in the substitution of tryptophan (TGG) by a stop codon (TGA) at amino acid position 263 (p.W263X). Verification of transmission of this mutation was done for all families members. This variant was absent even in the heterozygous state by direct sequencing in 100 Tunisian control chromosomes.

### Founder effect study

**Fig. 3:**
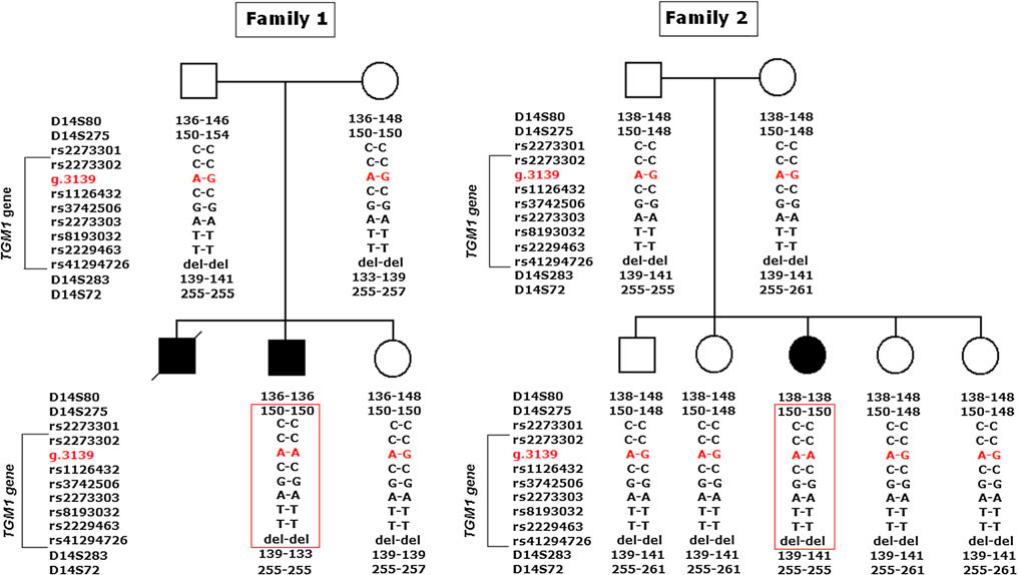
The pedigrees of the two Tunisian families showing the inheritance of the c.788G>A mutation. The same founder haplotype containing mutation is framed for the two patients. (*del* is a deletion of GGGGAGTCCAGGGCTCCCGGAG of rs41294726)

According to the prevalence of the c.788G>A mutation among our group of patients and since the studied families sharing the same mutation c.788G>A are all originated from south Tunisia, we test the hypothesis that this mutation arose on a founder ancestor in the distant past. For these reason, we analyzed four polymorphic genetic markers and explored 8 SNPs encompassing approximately 6 Mb in and around the *TGM1* gene to construct haplotypes for the mutant and normal *TGM1* chromosomes in the families. Patient’s haplotypes showed that the c.788G>A mutation has been transmitted in conserved bloc of 2 Mb. A conserved haplotype encompassing the markers D14S275–D14S283 was found (Fig. 3). This result revealed that the c.788G>A mutation is not the result of separate mutation occurring independently in different individuals, but is the result of a one-time mutation occurring in a common ancestor of all autosomal recessive congenital lamellar ichthyosis families in Tunisia.

## Discussion

In the present study, we report two Tunisian LI patients associated with nonsense c.788G>A mutation in the *TGM1* gene predicting an amino acid substitution of tryptophan for a stop codon (p.W263X). It introduces a premature stop codon after nearly one-third of the TGase-1 polypeptide and leads to a truncated enzyme lacking the majority of the active site. To the best of our knowledge, this homozygous mutation is not prevalent worldwide, being found in only two Tunisian LI patients [14, 18, 19] and suggested to be a recurrent mutation in Tunisian patients affected by severe LI.

The clinical phenotypes observed in our patients correspond to those that have been described as lamellar ichthyosis in the literature [20]. It is noteworthy that the previous Tunisian patients described by Esposito et al. and Hennies et al. [18, 19], additionally suffered from congenital hypothyroidism or from severe gluten sensitive enteropathy respectively. Moreover, Oji et al. [21] report a relatively mildly affected girl from France with compound heterozygous genotype c.788G>A/c.919C>G and abnormal in vivo TGase-1 activity in affected skin areas.

Several reports suggest a link between thyroid diseases and ichthyosis. Firstly, in patients affected by both autoimmune thyroid disease and acquired ichthyosis, thyroid hormone replacement resulted in improvement of ichthyosis [20, 22]. Moreover, TGase-1 deficient LI was also found to be associated to congenital hypothyroidism in a child of consanguineous parents [23]. In such case, ichthyosis and hypothyroidism could be co-inherited, since the *TGM1* and *TSHR* genes maps to the same chromosome localization (14q). This inter-familial variability, in clinical phenotype, encountered in LI patients sharing the same mutation could be due to differences in environmental factors or to the genetic background (influence of modifier gene) or both [24].

With regard to the nonsense nature of the c.788G>A mutation, two hypotheses may be evoked: either the translation of a truncated transglutaminase I protein lacking catalytic domain (core domain), or possibly the degradation of the nonsense mRNA that manifests as a complete absence of transglutaminase I protein. The most probable hypothesis is that the resulting nonsense mRNA is probably destroyed by the quality control surveillance mechanism NMD (nonsense-mediated mRNA decay) according to the PTC position rule (>50–55 nt upstream of the last exon–exon junction), which is detected in our patient at position c.788 [25].

Previously, using ^32^P labelled *TGM1* probes, mRNA was shown to be absent from cultured keratinocytes taken from a patient homozygous for the nonsense mutation R127X [26]. Northern blotting of tissue taken from a skin biopsy and cultured keratinocytes from a patient homozygous for the truncating mutation c.1297delT showed absent TGase-1 mRNA in vitro [27]. NMD, which decreases the amount of cellular mRNA with premature termination codons (PTCs), could be the mechanism that explains these observations [25].

High carrier rates are usually attributed to a founder effect in endogamic and consanguineous populations [28]. They are usually evidenced by conservation of haplotypes with directly associated markers. Since the c.788G>A mutation has not been described elsewhere, it seems to be specific to the Tunisian population. According to such finding and in agreement with the prevalence of this mutation reported in our study and the previous ones [18, 19], we demonstrated that a clustering of g.3139 G>A mutation in two Tunisian families is due to a common founder ancestor. In fact, the analysis of 4 microsatellite markers and 8 SNPs flanking or within the *TGM1* gene revealed a common haplotype carried by the patients sharing the same g.3139 G>A mutation. Hence, we suggest that this founder haplotype which ranges over 2 Mb could be the same for the two patients already reported and sharing the same mutation. In addition, the two families carrying the g.3139 G>A mutation are originated from southern Tunisia. Such rural communities are characterized by a high consanguinity and endogamy rates which supports our findings. Evidence for a founder effect of *TGM1* gene has been also reported in American and Spanish populations [2, 29]. Given that it was the only founder mutation described so far in Tunisia, the c.788G>A mutation could be the only variation responsible for LI in Tunisian population.

In summary, the conservation of a single haplotype surrounding the c.788G>A mutation suggested that this ancestor haplotype has a single origin. The finding of a founder effect in a unique recurrent mutation in a rare disease is essential for the implementation of protocols for genetic diagnosis, for genetic counselling of affected pedigrees and is fundamental to search for new therapies.
